# Thermochemical Evaluation of Banana Peel Biomass for Syngas Production: Thermal and Electrical Energy Production Potential

**DOI:** 10.1021/acsomega.5c06767

**Published:** 2025-11-21

**Authors:** Shirlene T. O. Santos, Deivson C. S. Sales, Adalberto F. Nascimento Júnior, Sergio P. R. Silva

**Affiliations:** † Departamento de Energia Nuclear, 28116 Universidade Federal de Pernambuco, Av. Prof. Luiz Freire 1000, Cidade Universitária, Recife, Pernambuco 50740-545, Brazil; ‡ Escola Politécnica de Pernambuco, 117110 Universidade de Pernambuco, Rua Benfica 455, Madalena, Recife, Pernambuco 50720-001, Brazil

## Abstract

The global use of fossil fuels remains high, leading to significant greenhouse gas emissions. To mitigate this, renewable energy sources must be explored, with biomass being a promising option. Biomass is carbon-rich and has a strong thermoconversion potential. In Brazil, banana peel stands out as an abundant biomass source suitable for this process. This study investigates the gasification of banana peel to produce syngas, which is a key precursor for synthetic fuels. The biomass was first prepared and characterized by analyzing moisture content, bulk density, and calorific value and through elemental, bromatological, and immediate analyses. Gasification was then performed at 600, 700, 800, and 900 °C. The results showed a high carbon content, especially volatile carbon, confirming its thermoconversion potential. Syngas rich in hydrogen was produced, with its concentration increasing with the temperature. The highest calorific values were observed at 900 °C, along with greater thermal and electrical energy potential. However, values at 800 °C were similar, suggesting that it is an optimal temperature for the process due to efficiency and potentially lower energy input requirements. This highlights banana peel’s viability as a feedstock for renewable energy through thermochemical conversion.

## Graphical abstract

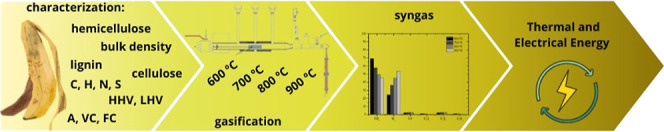

## Introduction

The world’s energy consumption still is based on fuels derived from petroleum, as well as natural gas and coal.^1^ As a consequence, a large amount of greenhouse gas emissions (GGE), such as CO_2_, NO_*x*_, and SO_*x*_, were produced,^2^ reaching 53.6 ± 5.2 GT CO_2_ e yr^–1^ in the past decade (2014–2023).^3^ In addition to contributing to environmental pollution, these energy sources also create a dependence on the oil market since most fuels are produced from this source.^4^

There are countries where the energy matrix is composed of approximately half of the share of clean sources and the other half of fossil fuel sources, such as Brazil, in which 47.4% of the domestic energy supply comes from renewable energy sources. In South American countries, the generation of electric power is dominated by hydroelectric plants, but the lack of useful volume in reservoirs can lead to a decrease in the level of generation during periods of scarcity. From the past decade onward, solar and wind power generation contributed significantly to the national energy production in these countries.^5^ However, because of international agreements, plants should be powered by renewable and clean fuels, such as biomass.^6^

Biomass is a renewable natural resource of carbon derived from organic materials, agricultural or industrial waste, which are composed of chemical energy content and can be presented in the form of solid, liquid, or gaseous biofuels.^7,8^ The bananas (*Musa* ssp) are the most consumed and produced fruit worldwide.^9^ In 2021, world banana production was 124 Mt,^10^ and an increase of 1.4% per year is estimated, reaching a production of 138 Mt in 2030.^11^ Brazil is the fourth largest producer in the world, with 6.8 Mt of bananas produced, occupying around 453,273 ha of harvested area.^10^

The peel is the major residue from bananas, being a very important biomass, representing 40% of the fruit’s weight.^12^ In 2021, around 49.6 Mt of banana peels were generated, resulting in large amounts of waste. Tons of this waste are generated daily, mainly in homes, coffee shops, restaurants, fairs, and industries.^13^

The banana peel is a biomass rich mainly in lignin, cellulose, hemicellulose,^14^ proteins, and phenolic compounds.^15^ Due to the presence of these compounds, especially lignin, the natural degradation of banana peels does not occur easily and quickly. Because of the organic load, these residues, in addition to not contributing economically in a positive way, can also cause phytosanitary problems such as the proliferation of pathogenic animals, environmental pollution, and unpleasant odors due to the released gases such as sulfides, ammonia, and methane, as most of this waste is disposed of without any form of treatment.^16^ These residues can also be used as organic fertilizer and animal feed due to their low tannin content and high fiber content.^13^ In addition, there is a lot of fruit loss even before it reaches consumers, which can represent up to 30% of waste from planting to commerce.

The banana peel can be directed to the generation of thermal and electric energy through thermochemical routes for the production of solid, liquid, and gaseous biofuels.^17,18^ Among the available thermochemical routes, biomass gasification can be considered the most efficient and economical when the conversion of lignocellulosic raw material into renewable energy is desired. Gasification is an energy conversion route that occurs in the presence of oxidizing agents, under substoichiometric conditions, with the purpose of transforming solid fuels (or liquids) into gaseous fuels, with sufficient energy power to provide thermal or electrical energy,^19^ avoiding the emission of toxic gases.^20^

The most used biomass in thermoelectric plants in Brazil is sugarcane bagasse, which has a heating value of 15–19 MJ kg^–1^ ^21^ and contributes 15.4% to the internal energy supply,^5^ while the banana peel has a heating value of 22 MJ kg^–1^,^17^ thus becoming a viable alternative, in relation to the energy produced in combustion, as an alternative source of energy, mainly in the Northeast region of the country. Brazil, where greater stability can occur in the energy matrix due to periods of water scarcity and, consequently, the reduction of hydroelectric reservoirs. In this sense, the study of the thermochemical processing of banana peels to determine the potential energy production, mainly electrical, is innovative and has been little explored.

In this sense, in the present work, the banana peel biomass was characterized and gasified for hydrogen-rich syngas production using a batch mode, which operates in a fixed bed, aiming for a subsequent use in the generation of thermal or electric energy.

## Methodology

### Preparation

The experiments used the biomass of Pacovan banana peels belonging to the silver subgroup obtained from homes and snack bars in the city of Recife, state of Pernambuco—Brazil. The biomass was collected in plastic bags, stored in hermetically closed containers, and refrigerated at 4 °C until use. The peels were previously dried at room temperature (25 °C) for 6 h to allow for the evaporation of the free water. To ensure a completely dry biomass, the residue was placed in an oven (brand: Quimis; model: Q-317B222; temperature: 110 °C) until it reached a constant mass.^22^ After drying, the peel was ground in a knife mill (brand: MARCONI; model: MA-48) and passed through granulometric sieves. The samples that remained on the sieves between 20 and 24 Mesh (850 and 710 mm/μm, respectively) were subjected to the quartering technique. The choice of particle diameter was based on the work of Dias et al.^23^ Finally, the selected sample was stored in a hermetically closed glass container at room temperature (25 °C) for further analysis.

The banana peel is an agricultural waste; therefore, its physicochemical characteristics depend on specific factors such as cultivated species, climate, harvesting time, ripening stage, and soil characteristics, among others.^24^ The characterization results presented in this study were obtained by averaging the analyses that were performed in triplicate.

### Determination of Moisture Content

The determination of the moisture content was carried out by two methods: initially, the moisture content was measured in the sample in natura before any drying process. Then a new moisture content was measured in the samples that had undergone only drying at room temperature under sunlight (32 ± 5 °C). In both methods, the ABNT NBR 14.929:2017 ^25^ standard was used. The initial mass of the peel was measured on a calibrated analytical balance (brand: Shimadzu; model: AY220), and it was later placed in an oven at 110 °C until a constant mass was achieved, ensuring that the biomass was on a dry basis. The moisture content (*w*) (%) was determined by eq 1.^25^

1 $$w=100\left(1-\frac{m_{s}}{m_{u}}\right)$$

where *m*_s_ (g) and *m*_u_ (g) are the masses of the sample before and after the drying process, respectively.

### Determination of Bulk Density

The determination of bulk density (*d*_g_) (kg m^–3^) was based on the ASTM D5057-10.^17^ In a graduated cylinder (brand: Uniglas; volume: 10 mL), and the mass of dry biomass (*m*_b_) (g) was added until the cylinder was filled to the volume (*V*_b_) (10 mL). In sequence, this mass was weighed on an analytical balance (brand: Shimadzu; model: AY220). The bulk density (*d*_g_) was determined by the *m*_b_/*V*_b_ ratio.

### Elemental Analysis

The elemental composition expresses the quantity of the main constituent elements of a biomass: carbon, hydrogen, nitrogen, oxygen, and sulfur. The percentage in proportion of these elements was determined using a CHNOS Elemental Analyzer (brand: ELEMENTAR; model: Vario Macro Cube), according to ASTM D5373 standards.^26^ The percentage of oxygen is not provided by the equipment, but it can be calculated by the difference in the other elements. The carbon content was determined by combining the organic and inorganic ones. The hydrogen content is due to the presence of hydrogen in the biomass and residual from water.

### Determination of Heating Values

The heating values of biomass were determined using an automatic digital calorimeter (brand: IKA; model: C2000), following the ASTM D240.^26^ The amount of energy produced by direct and complete combustion was determined. The samples used in the experiments were on a dry basis and compacted in cylindrical pellet formats (length: 3.0 mm; diameter: 1.3 mm). The pellet (400 mg) was placed in the adiabatic bomb and reacted with oxygen in excess using the dynamic method with an initial temperature of 25 °C. The ignition was performed using platinum resistance and transferred to the pellet through a cotton wick. This first result was determined by the equipment as the higher heating value (*HHV*_biomass_) (MJ kg^–1^). The water that condensed inside the bomb (combustion water) was collected in a 250 mL Erlenmeyer flask and later titrated with 0.1 N sodium hydroxide (molecular formula: NaOH; brand: Dinâmica) solution using sodium sulfamethazine (molecular formula: C_14_H_14_N_3_NaO_3_S; brand: Dinâmica) as an indicator. A volume of 20 mL of 0.05 N sodium carbonate (molecular formula: Na_2_CO_3_; brand: Dinâmica) solution was added to the Erlenmeyer flask, which was then titrated again with 0.1 N hydrochloric acid (molecular formula: HCl; brand: Dinâmica) solution. From the volumes of HCl and NaOH used, the second result was determined by the equipment as the lower heating value (*LHV*_biomass_) (MJ kg^–1^).

### Bromatological Analysis

The quantities of cellulose, hemicellulose, and lignin present in the biomass were determined using the methodology presented by Hall and Mertens.^27^ For neutral detergent fiber percentage (NDF) (%) determination, 1 g of dried banana peel (710 mm/μm ≤ granulometry ≤850 mm/μm) was mixed with 100 mL of neutral detergent solution previously prepared using 30 g of sodium lauryl sulfate (molecular formula: C_12_H_12_NaSO_4_; brand: Dinâmica), 10 mL of triethylene glycol (molecular formula: C_6_H_14_O_4_; brand: Anidrol), 6.81 g of sodium tetraborate (molecular formula: Na_2_[B_4_O_5_(OH)_4_]·8H_2_O; brand: Neon), 4.61 g of sodium phosphate (molecular formula: Na_3_PO_4_; brand: Dinâmica; purity: 99.9%), and 18.61 g of ethylenediaminetetraacetic acid (acronym: EDTA; molecular formula: C_10_H_16_N_2_O_8_; brand: Dinâmica) in 1 L of solution. In addition, 15 drops of amyl alcohol (molecular formula: C_5_H_12_O; brand: Anidrol; purity: 99.9%) were added to the solution to avoid the formation of foams due to the presence of the EDTA. The system was taken to a fiber digester block (brand: MARCONI; model: MA450/6) for 1 h at reflux and constant temperature until it reached a boiling point. Then, using porous crucibles, the biomass was filtered using an evacuation pump (brand: New Pump; power: 200 W). To ensure the removal of any residual impurity, 10 mL of acetone (molecular formula: C_3_H_6_O; brand: Anidrol; purity: 99.9%) was added to wash the sample. The mass deposited in the crucible represents the amount of cell wall constituents: cellulose, hemicellulose, lignin, and insoluble ash, since the procedure removed soluble ash and other constituents. The mass, after drying in an oven (brand: Quimis; model: Q-317B222) at 110 °C for 2 h, was weighed on an analytical balance (brand: Shimadzu; model: AY220). The NDF value was determined according to eq 2.^27^

2 $$NDF=100\left(\frac{m_{aNDF}}{m_{db}}\right)$$

where *m*_aNDF_ (g) is the mass of the sample after digestion for NDF and *m*_db_ (g) is the mass of the banana peel on a dry basis.

The acid detergent fiber percentage (ADF) (%) determination was based on Kamal et al.^28^ For determination, 1 g of dry biomass was ground (similar to NDF determination) and placed in a beaker to react with a solution prepared with 1 L of 0.1 N sulfuric acid (molecular formula: H_2_SO_4_; brand: Química Moderna; purity: 99.9%) and 20 g of cetyltrimethylammonium bromide (acronym: CTAB; molecular formula: C_19_H_42_BrN; brand: Anidrol). In addition, 15 drops of amyl alcohol (molecular formula: C_5_H_12_O; brand: Química Moderna; purity: 99.9%) were added to the solution to avoid the foam due to the presence of CTAB. The set was refluxed in the digester block for 1 h; at the end, it was filtered and washed with 10 mL of acetone (molecular formula: C_3_H_6_O; brand: Anidrol; purity: 99.9%). The ADF value was determined according to eq 3.^28^

3 $$ADF=100\left(\frac{m_{aADF}}{m_{db}}\right)$$

where *m*_aADF_ (g) is the mass of the sample after digestion for NDF.

For Klason lignin percentage (KL) (%) determination, 300 mg of the sample of biomass was macerated with 3 mL of sulfuric acid (molecular formula: H_2_SO_4_; brand: 72% v/v) for 1 h, resulting in a homogeneous mixture. The content was transferred to a beaker with 84 mL of distilled water and then placed in a fiber digester block (brand: MARCONI; model: MA450/6) for 4 h. The contents were filtered in a porous crucible and taken to dry in an oven (brand: Quimis; model: Q-317B222). The KL value was determined according to eq 4.^29^

4 $$KL=100\left(\frac{m_{l}}{m_{db}}\right)$$

where *m*_l_ (g) is the mass of the sample after digestion using H_2_SO_4_.

In the determination of total ash percentage (TA) (%), the banana peel sample on a dry basis was weighed using an analytical balance (brand: Shimadzu; model: AY220) and then calcined in an oven (brand: Quimis; model: Q-317B222) at 775 °C for 2 h. After cooling to room temperature (25 °C), the sample was weighed again. The mass of total ash of the sample (*m*_TA_) (g) was the difference between initial and final mass. The TA value was determined according to eq 5.^30^

5 $$TA=100\left(\frac{m_{TA}}{m_{db}}\right)$$

In the determination of insoluble ash percentage (IA) (%), the total ashes were diluted in excess distilled water and then filtered using a funnel and filter paper (weight: 80 g m^–2^). From the ashes retained on the filter paper after drying in an oven (brand: Quimis; model: Q-317B222) at 110 °C for 2 h, the IA value was determined by eq 6.^30^

6 $$IA=100\left(\frac{m_{IA}}{m_{TA}}\right)$$

where *m*_IA_ (g) is the mass of insoluble ash. The soluble ash percentage (SA) (%) was determined by the difference between the TA and IA values. The real insoluble ash percentage (RIA) (%) was determined according to eq 6.^30^

7 $$RIA=100\left(\frac{m_{IA}}{m_{db}}\right)$$

The hemicellulose (HCLS) (%) and cellulose (CLS) (%) percentages were determined by eqs 8 and 9, respectively.^27^

8 HCLS=NDF−ADF

9 CLS=ADF−KL−RIA

### Immediate Analysis

The immediate chemical composition was determined using a thermogravimetric balance (brand: Shimatzu; model: DTG-60) according to ASTM E790/830/897.^26^ In the process, the biomass was combusted with oxygen under stoichiometric conditions at a heating rate of 10 min^–1^ up to 900 °C under an inert atmosphere of nitrogen. The ash (A) (%), volatile carbon (VC) (%), and fixed carbon (FC) (%) percentages were determined.

### Evaluation of Biomass Gasification

For the gasification experiment, a batch-mode gasifier was used, which operates in a fixed-bed, updraft, and countercurrent flow mode with indirect heating provided by electric resistances. In Figure 1 is shown a schematic drawing of the reactor according to Peres et al.^31^ The biomass was deposited into a stainless-steel crucible AISI 304, and then the reaction was performed. In Table 1 are shown the conditions used in the experiments.

**Figure 1:**
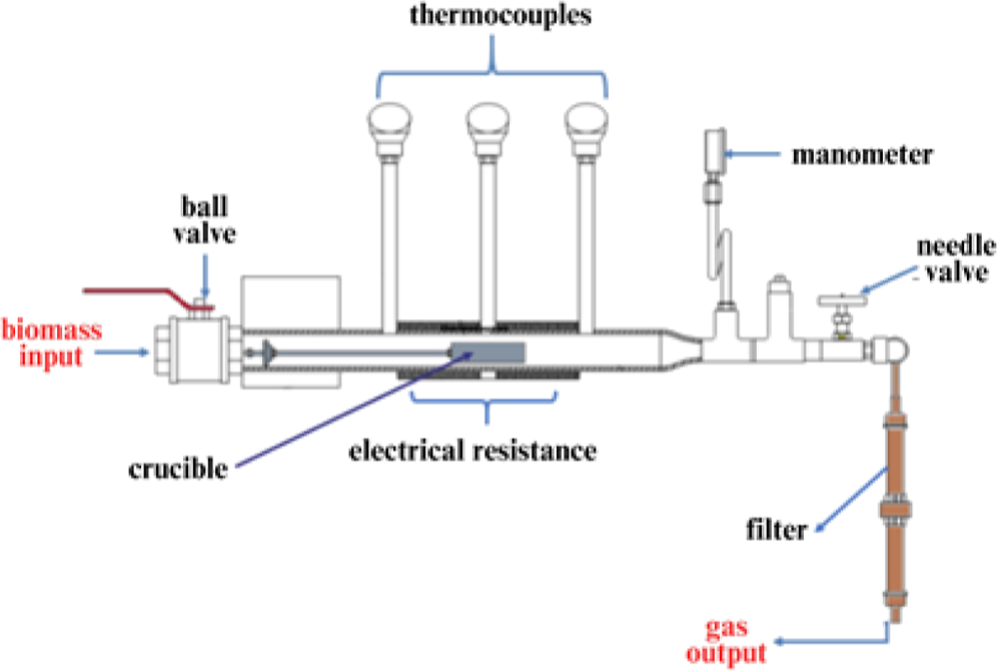
Scheme of the batch-mode gasifier.

**Table 1:** Operational Conditions

Parameter	Value
Residence time (min)	5.0
Mass of biomass (g)	2.0–5.0
Particle size (mesh)	24
Gasifying agent	Air
Flow rate (mL min^–1^)	100
Equivalence ratio	0.25
Temperature (°C)	600; 700; 800; 900

After the working temperature was reached, the crucible containing the sample was placed inside the gasifier. Subsequently, the entrance to the equipment was closed, thus creating a controlled environment. The gas outlet was kept closed during the reaction to allow pressure measurement. At the end of the reaction, the outlet was opened, and the gaseous mixture produced passed through a filter (steel wool + sawdust) to retain water vapors, thus avoiding excessive humidity. After this process, the gas mixture was analyzed. Between each experiment, the gasifier was cleaned and purged with compressed air.

The gaseous mixture was analyzed using two gas chromatographs equipped with a thermal conductivity detector (TCD), since hydrogen was quantified separately. For hydrogen analysis, a first gas chromatograph (brand: SRI Instruments; model: 8610C; volume injected: 500 μL; column: 1/8 in. × 6 ft 5A molecular sieve packed columns; carrier gas: argon; oven temperature: 40 °C; detector temperature: 200 °C; current: 125 mA) was used, while for analysis of other gases, a second chromatograph (brand: Thermo Fisher Scientific Inc.; model: TRACE GC Ultra; volume injected: 1:10 split/splitless; column: 15 m × 0.32 mm × 1 μm Carbon PLOT; carrier gas: hydrogen; oven temperature: 40 °C; detector temperature: 200 °C; voltage: 5 V) was used.

The analysis conditions were established based on the expected products, in terms of the main reactions involved in biomass pyrolysis: partial combustion of carbon (C + 0.5O_2_ → CO), Boudouard (C + CO_2_ ↔ 2CO), methanation (C + 2H_2_ ↔ CH_4_), and water–gas shift (WGS) (CO + H_2_O ↔ CO_2_ + H_2_), in addition to higher hydrocarbons.^32^

### Determination of Thermal Efficiency of Gasification and Carbon Conversion Efficiency

The thermal efficiency of gasification (TEG) (%) was determined according to eq 10.^18^

10 $$TEG=100\left(\frac{LHV_{syngas}\xi_{syngas}}{LHV_{biomass}}\right)$$

where LHV_syngas_ (MJ m^–3^) is the lower heating value of the syngas, ξ_syngas_ (m^3^ kg^–1^) is the syngas yield, and LHV_biomass_ (MJ kg^–1^) is the lower heating value of the biomass.

Considering the research developed by,^33^ the gas mixture is considered to be composed of ideal gases (ideal gas mixture), and the volume of gas produced (*V*_syngas_) (m^3^) was determined by ideal gas equation using the pressure and temperature measured during the process.

The syngas yield (ξ_syngas_) was determined by eq 11.^18^

11 $$\xi_{syngas}=\frac{V_{syngas}}{m_{b}}$$

where *m*_b_ (kg) is the mass of the biomass.

The lower heating value of the syngas (LHV_syngas_) was determined according to eq 12.^34^

12 $$LHV_{syngas}=\sum \phi_{i}LHV_{i}$$

where ϕ_*i*_ is the volume fraction obtained by chromatography, and LHV_*i*_ (MJ kg^–1^) is the lower heating values of component *i* (*i* = H_2_, CO, CH_4_, or C_*n*_H_*m*_) in the gas mixture.

The carbon conversion efficiency (CCE) (%) was determined by eq 13.^4,35^

13 $$CCE=100\left[\frac{12\xi_{syngas}\left(\phi_{CO}+\phi_{{CO}_{2}}+\phi_{{CH}_{4}}\right)+2\sum C_{n}H_{m}}{22.4\phi_{C}}\right]$$

where ϕ_C_ is the volume fraction of carbon determined by the elemental analysis.

### Estimative of Thermal and Electrical Energy Potential

The thermal (*E*_T_) (kWh) and electrical (*E*_E_) (kWh) energy potential produced by the conversion process were determined by eqs 14 and 15, respectively.^36^

14 $$E_{T}=0.277\eta_{TE}E_{CEP}m_{b}$$

15 $$E_{E}=0.277\eta_{EE}E_{CEP}m_{b}$$

where *E*_CEP_ (MJ kg^–1^) is the chemical energy produced from syngas; η_TE_ and η_EE_ are the efficiencies of converting chemical energy to thermal and electric energies, respectively. The values of η_TE_ and η_EE_ were 0.500 and 0.345, respectively, according to the Sankey diagram.^37^

The chemical energy produced from syngas (*E*_CEP_) was determined according to eq 16.^36^

16 $$E_{CEP}=\frac{V_{syngas}LHV_{syngas}}{m_{b}}$$

## Results and Discussion

### Determination of Moisture Content

The biomass peel yield was 41.8%, indicating the percentage of waste produced by the number of fruits. This value is similar to that obtained by Anniwaer et al.,^4^ who obtained values of 30–40% (m/m). The moisture value of fresh banana peels was 74.44%, similar to that obtained by He et al.^20^ For sugarcane bagasse, which is a biomass widely used in ovens and boilers for the production of thermal and electrical energy, the typical moisture content is 50.73%.^38^ The peels dried at room temperature under sunlight (32 ± 5 °C; predrying step) presented 9.8% moisture content, indicating that this is an efficient step for removing water present in the biomass.

### Determination of Bulk Density

The bulk density of the biomass was 520 kg m^–3^, a value similar to 600 kg m^–3^ obtained by Duangkham and Thuadaij^17^ also for banana peel. The low value of bulk density observed was influenced mainly by high moisture content (74.44%), which directly affected the direct combustion, making it unstable and incomplete, thus producing a large amount of gaseous pollutants. In this sense, this biomass is indicated for thermal conversion, such as gasification, as indicated by Cardona et al.^39^

### Elemental Analysis

The results of the elemental analysis of the biomass are listed in Table 2. The results obtained by other authors were included for comparison purposes.

**Table 2:** Elemental Composition

Biomass	C (%)	N (%)	H (%)	S (%)	O (%)	ref.
Banana peel	48.00	1.10	6.65	0.29	43.96	This work
Banana peel (Ecuador)	44.30	1.30	4.90	-	49.50	^4^
Banana peel	43.71	1.46	5.66	0.07	40.87	^16^
Wood	45.70	1.89	7.57	1.01	56.20	^40^
Sugar cane bagasse	48.64	0.16	5.87	0.04	42.85	^32^

The C is the main element used for thermal conversion of biomass in syngas. Since the N content (1.10%) in biomass was low, there was a minor formation of N_2_ and NO_*x*_ below that observed for fossil fuels.^41^ Similar behavior is expected for SO_*x*_ formation, since the S content (0.29%) also was low. During the gasification, H plays an important role in the final composition of syngas due to participation in the water gas shift reaction, producing H_2_. On the other hand, the O content was similar to that observed by other authors; however, it presented a high value compared to other elements related to the presence of cellulose, hemicellulose, and lignin in the biomass.^42^

Based on Table 2, the balanced chemical equation was determined as presented in eq 17.

17 $$C_{10}H_{17}O_{7}+10.75\left(O_{2}+3.76N_{2}\right)\to 10{CO}_{2}+8.5H_{2}O+40.42N_{2}$$

### Determination of Heating Values

The heating values for biomass are shown in Table 3. Furthermore, the results obtained by other authors were also included for comparison purposes. The HHV_biomass_ value observed in this work was similar to those obtained by other authors; however, the LHV_biomass_ was higher (except for mango seed). According to Miranda et al.,^21^ biomasses with a calorific value between 15 and 19 MJ kg^–1^, as used in this work, have great potential for use as industrial biofuel. The elements that most interfere with the calorific value are C, O, and H. The first two react in combustion exothermically, producing CO_2_ and H_2_O, and thus the high C/H ratio (7.22%) contributed satisfactorily to the calorific power.

**Table 3:** Heating Values

Biomass	HHV_biomass_ (MJ kg^–1^)	LHV_biomass_ (MJ kg^–1^)	ref.
Banana peel	19.00	18.70	This work
Banana peel	16.12	-	^16^
Charcoal	22.00	-	^17^
Firewood	18.30	16.90	^43^
Empty bunches of bananas	-	9.91	^39^
Citrus peel	17.80	16.60	^44^
Mango seed	19.70	18.41	^26^

### Bromatological Analysis

The results of bromatological analysis for biomass are shown in Table 4. The results obtained by other authors also were included for comparison purposes. The results indicated a high percentage of lignin for banana peel compared to other authors, indicating the recalcitrance and stability of this biomass, since one of its functions is to combat oxidative stress and protect the fruit pulp from external attacks such as the action of microorganisms.^2,45^ The percentage of 45.8% lignin agrees with the results obtained from,^14^ who observed a high lignin content in banana peels, finding values of 37.4 ± 0.6%. The amounts of cellulose, hemicellulose, and lignin obtained in this study were similar to those observed by authors who used a similar species of biomass.^4,14^

**Table 4:** Bromatological Analysis

Biomass	CLS (%)	HCLS (%)	KL (%)	ref.
Banana peel	10.94	9.72	45.80	This work
Banana peel	23.30	15.60	37.40	^14^
Banana peel	7.04	7.64	9.70	^4^
Sugar cane bagasse	31–54	13–39	11–27	^21^
Wood (Padouk)	40.80	11.10	29.00	^19^
rice straw	31–47	19/27	5–24	^7^

### Immediate Analysis

The results of the immediate analysis for the biomass are shown in Table 5. The results obtained by other authors also were included.

**Table 5:** Immediate Analysis

Biomass	A (%)	VC (%)	FC (%)	ref.
Banana peel	6.68	91.11	2.12	This work
Banana peel	9.70	66.10	24.2	^4^
Banana peel	0.50	94.00	0.70	^15^
Empty bunches of bananas	5.24	59.30	19.29	^39^
Sugar cane bagasse	1.94	74.98	13.57	^46^
ango seed	2.57	73.92	-	^26^

According to the immediate analysis, volatile carbon represented around 91.11%, indicating the amount of biomass matter available to undergo thermoconversion processes. The higher the volatile carbon value, the greater is the conversion of the banana peel into combustible gases. The inorganic matter represented by ash was considered as a residue after the oxidation of the biofuel, as its presence reduces the calorific value, and the accumulation of mass can cause incrustation in pipes and increased corrosion; that is, its content is unwanted. For this study, 6.68% of ash was found, which is a high percentage when compared to more traditional biomasses such as sugar cane bagasse, which is around 1.94%,^46^ but it is still a lower percentage than the 9.7% found in the banana peel study by a previous study,^4^ and the 9.84% of ash observed by Islam et al.^2^ The high amounts of ash in this type of biomass can be explained by the presence of inorganic elements, as each 100 g of banana peel on a dry basis contains around 1202.4 mg of calcium; 291.66 mg of magnesium; 800 mg of potassium; 250.93 mg of phosphorus; 784.56 mg of zinc and 558.5 mg of iron.^2^

The fixed carbon of 2.12% indicates the amount of matter that remains burning; the lower the amounts of FC, the lower the production of residual biochar after gasification. The banana peel used was satisfactory due to the low amount of FC, especially when compared to the banana peels studied by Anniwaer et al.^4^ and bagasse studied by Anukam et al.^46^ The low percentage of FC is also found in banana peels analyzed by Verma and Mishra.^15^

### Evaluation of Biomass Gasification

The results of the evaluation of biomass gasification are shown in Figure 2. The production of syngas (CO, H_2_) and several hydrocarbons (CH_4_, C_2_H_4_, C_2_H_6_, and C_3_H_8_) was observed. The increase in temperature was associated with a decrease in the concentration of CO, different from that observed for CH_4_. The percentages of CH_4_ produced were higher at all temperatures due to the conversion of C and CO_2_ into CO and, consequently, CO into H_2_ into CH_4_ (reverse methane steam reforming), as observed by Gao et al.^47^ In addition, the other hydrocarbons formed can also decompose during the gasification process into H_2_ and CO, further increasing the concentration of these products.^47^

**Figure 2:**
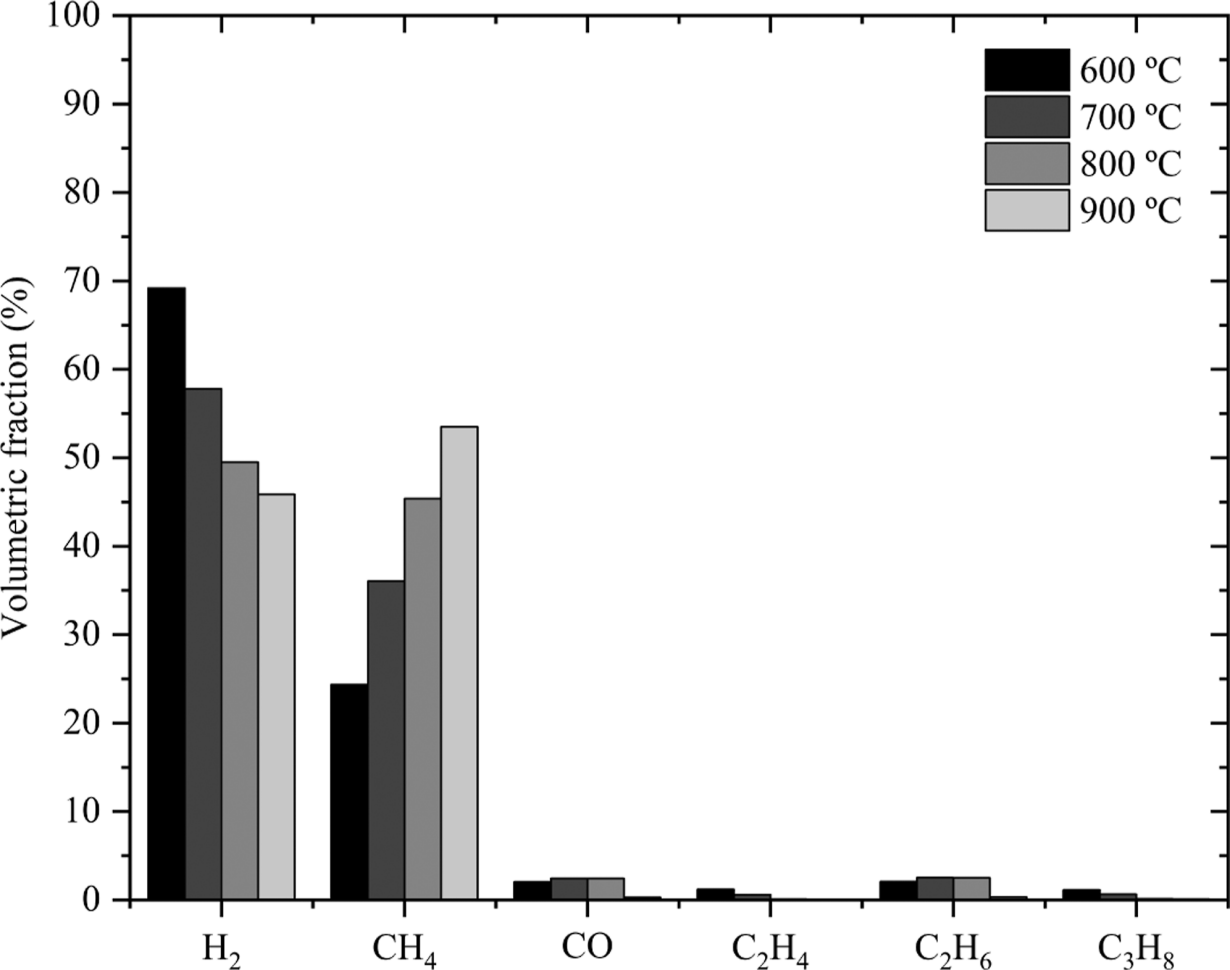
Volumetric fraction of the syngas.

The percentage of H_2_ decreases as temperature increases. The Boudouard reaction is favored under these conditions since its free energy is less than the water gas shift reaction (WGSR), in which H_2_ would be produced. The increase in temperature, especially above 700 °C, directed the WGSR in the direction of the reactants, inhibiting the formation of H_2_ and CO.^47^ From 700 °C onward, the percentages of hydrocarbons are practically constant, with C_2_H_4_ varying between 0.58 and 0.04%, C_2_H_6_ between 2.51 and 0.3%, and C_3_H_8_ between 0.64 and 0.08%. These low yields in relation to other gases indicate less relevant oxidation reactions, as observed by Cardona et al.^39^ Furthermore, the gasifying agent flow rate used in the present work (100 mL min^–1^) was lower than those observed in the literature (>300 mL min^–1^) for similar residence times (∼5 min),^20,39,47,48^ resulting in a slower pyrolysis reaction that allows the breakdown of heavier hydrocarbons formed into lighter ones, favoring the production of CH_4_.

### Determination of Thermal Efficiency of Gasification and Carbon Conversion Efficiency

The lower and higher heating values of the syngas for different temperatures are shown in Figure 3. The increase in the temperature resulted in an increase in both HHV_syngas_ and LHV_syngas_. The LHV_syngas_ was favored at the highest temperature due to the occurrence of carbon gasification and methane steam reforming reactions (endothermic reactions). In this sense, it is necessary to provide enough energy (which justifies the better process running at higher temperatures) to overcome the high activation energies of the reactions so that biomass gasification can then occur, as observed by Chang et al.^32^

**Figure 3:**
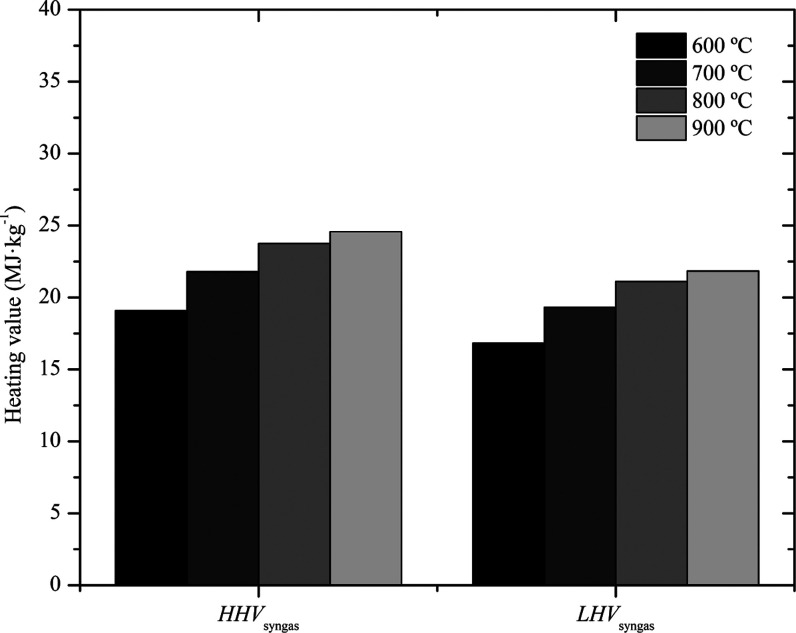
Lower and higher heating values of the syngas.

Although the LHV_syngas_ at a temperature of 900 °C was the highest value obtained, at 800 °C the result was very similar, as also observed by Lim et al.^29^ The authors explain that LHV_syngas_ above 900 °C tends to decrease regardless of the gasification agent. Therefore, it is preferable to choose gasification at a temperature of 800 °C due to the reduction in energy expenditure for the formation of syngas. At the lowest gasification temperature (600 °C), the lowest HHV_syngas_ and LHV_syngas_ were obtained because, as the biomass presented high lignin values (45.8%), a higher temperature is necessary for decomposition. A higher temperature provides higher gas pressure, resulting in greater syngas volume, due to greater conversion of carbon into gaseous products, as reported by Sikarwar et al.^49^

In Figure 4 are shown values of the thermal efficiency of gasification (TEG) and carbon conversion efficiency (CCE) at different temperatures.

**Figure 4:**
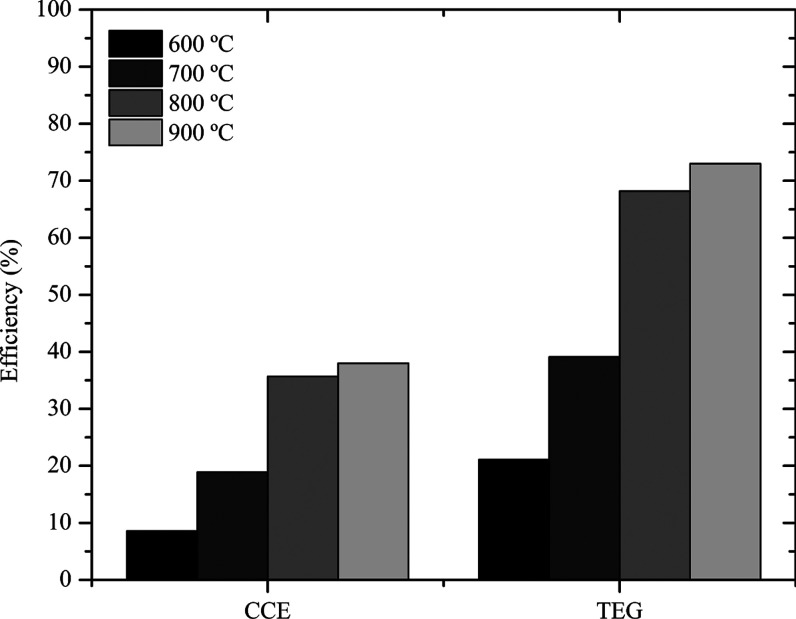
Thermal efficiency of gasification (TEG) and carbon conversion efficiency (CCE) values at different temperatures.

From the experiments, it was observed that volatilization of the components occurred more satisfactorily with increasing temperature, which directly influenced the improvement of TEG and CCE, contributing to the reduction of charcoal production, since the available carbon from biomass ends up being converted mainly into CO_2_, CH_4_ and CO. According to Anniwaer et al.,^4^ the increase in temperature accelerates the transfer of heat and mass between particles, benefiting their breakage, in addition to gasification reactions (mainly endothermic ones).

At 900 °C, the highest percentages of TEG and CCE were obtained, and a lower amount of biochar (24.0%), which in this case is an unwanted coproduct. These values agree with Anniwaer et al.^4^ who also observed higher CCE values, using the same biomass, with increasing temperature, and with He et al.,^20^ which at 850 °C obtained 41.9% of CCE for dried banana peels. At 600 °C, the lowest CCE rate (8.6%) and highest biochar production (32.8%) was observed. The TEG presented the most significant difference as the temperature changed since at the highest temperature it is almost four times greater than at the lowest temperature.

### Estimative of Thermal and Electrical Energy Potential

In Figure 5 is shown the chemical energy produced from syngas. The lowest chemical energy produced (79.9 MJ) was observed in gasification at 600 °C, while at temperatures of 800 and 900 °C, there were similar results of 256 and 275.9 MJ, respectively. Similar values were obtained by Xiong et al.^48^ above 800 °C.

**Figure 5:**
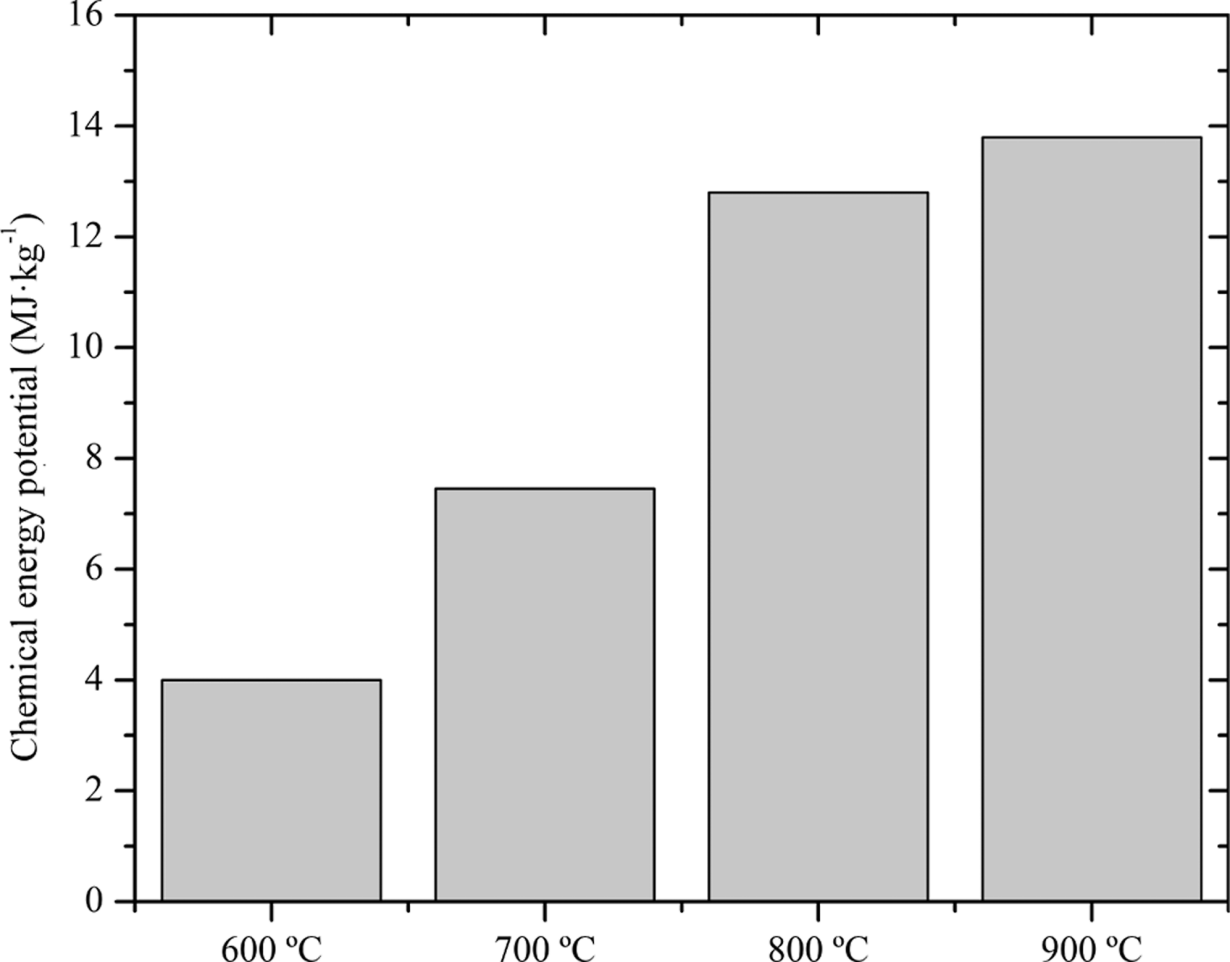
Chemical energy potential of the syngas.

In Figure 6 is shown the thermal (*E*_T_) and electrical (*E*_E_) energy potential produced by the conversion process.

**Figure 6:**
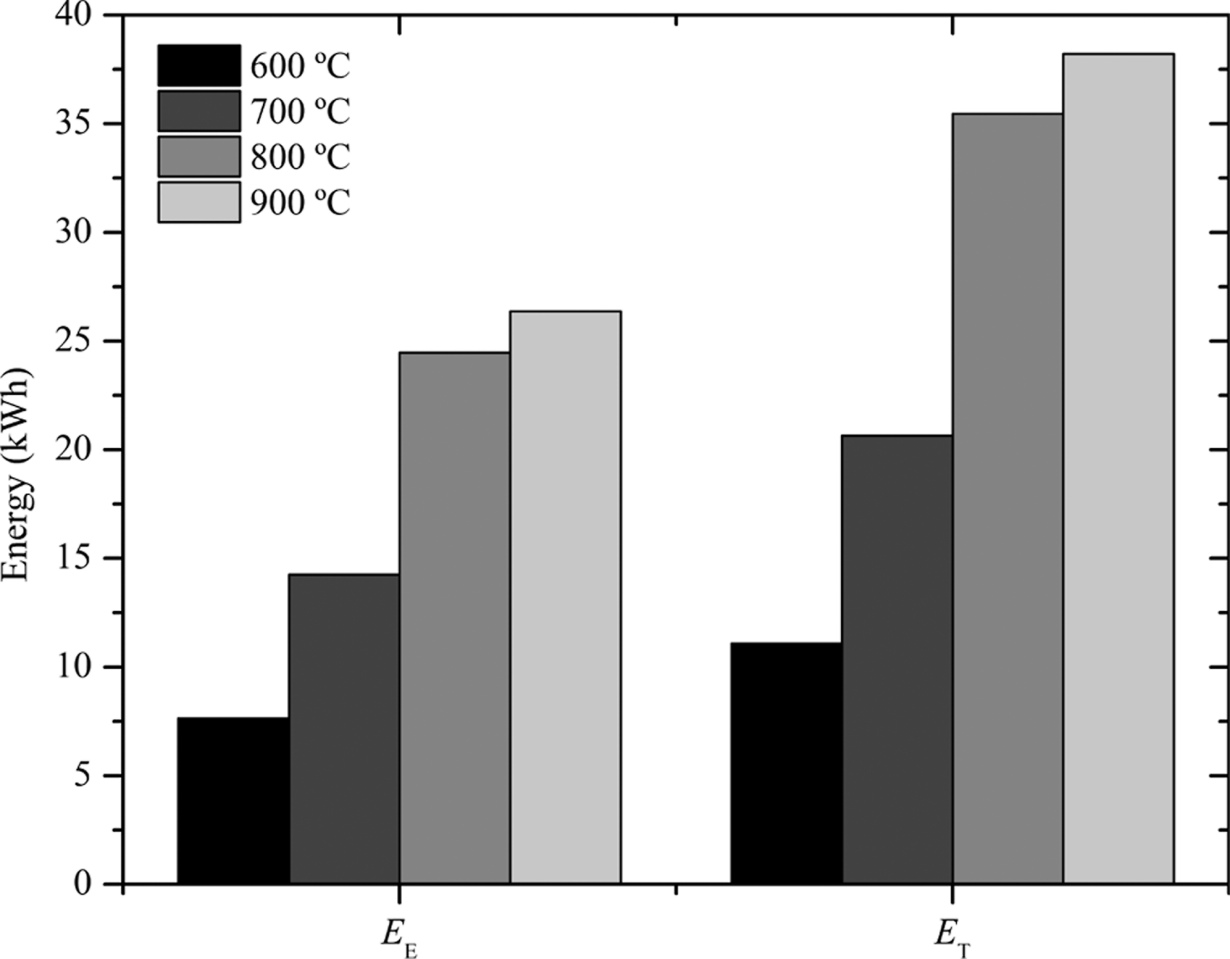
Energy potential of the syngas.

The results indicated the generation of 26.37 kWh of *E*_E_ and 38.21 kWh of *E*_T_ at 900 °C. In this sense, the increase in temperature also favored the production of these energies. At 800 °C,^39^ obtained 1.1 kWh kg_biomass_^–1^, while in the present work at the same temperature, 1.2 kWh kg_biomass_^–1^ was obtained, as well as 1.3 kWh kg_biomass_^–1^ at 900 °C. In general, the results indicated that the gasification process can occur at 800 °C, since no significant increase was observed that would justify the increase in temperature by 100 °C.

The Brazilian annual electrical energy generation, considering the quantity of banana peels produced in the country, can be estimated based on the results obtained by gasification of biomass studied in the present work. The Brazilian electrical generation from banana peel biomass is expected to be 3.6 GWh year^–1^ (based on 800 °C results) while this value worldwide is GWh year^–1^. In 2021, Brazilian electrical generation from biomass (wood, sugarcane bagasse, biodiesel, among others) was 52.2 GWh, indicating that banana peel biomass presents great potential for growth in its use for energy generation.

## Conclusions

In the present work, the potential of banana peel biomass (agro-industry or urban waste) for the generation of bioenergy by gasification was investigated. The characterization of biomass indicated 74.44% of moisture, 520 kg m^–3^ of bulk density, and mainly 91.11% of VC, indicating high tendency for thermoconversion. The biomass gasification produced syngas in quantities of 0.13 to 0.21 m^3^ kg^–1^ of dry biomass, with a lower heating value between 16.83 and 21.84 MJ m^–3^ at temperatures from 600 to 900 °C. The production of electrical and thermal energy increased as the temperature increased, reaching values of 26.37 and 38.21 kWh at 900 °C; however, it was observed that similar values were obtained at 800 °C, indicating that this is a more suitable temperature for the process. The use of banana peel as a raw material for energy production adds value to waste that is generally discarded, as well as helping to reduce the consumption of nonrenewable fuels. On the other hand, it is important to highlight that the plan to harness this potential is related to the implementation of an efficient biomass sorting and storage system, as well as the installation of gasification plants close to these locations. As suggestions for the continuation of this research, it is important to investigate the variation in the equivalence ratio, the possibility of cogeneration with other biomasses, and different air flow rates.
